# Ene-reductase transformation of massoia lactone to δ-decalactone in a continuous-flow reactor

**DOI:** 10.1038/s41598-021-97585-w

**Published:** 2021-09-22

**Authors:** Ewa Szczepańska, Danilo Colombo, Francesca Tentori, Teresa Olejniczak, Elisabetta Brenna, Daniela Monti, Filip Boratyński

**Affiliations:** 1 Department of Chemistry, Wroclaw University of Environmental and Life Sciences, Norwida 25, 50-375 Wrocław, Poland; 2 Dipartimento di Chimica, Materiali ed Ingegneria Chimica “Giulio Natta” Politecnico di Milano, Via Mancinelli 7, 20131 Milan, Italy; 3 Istituto di Scienze e Tecnologie Chimiche “Giulio Natta” (SCITEC)-CNR, Via Mario Bianco 9, 20131 Milan, Italy

**Keywords:** Biochemistry, Biotechnology

## Abstract

The demand for natural food flavorings increases every year. Biotransformation has become an attractive approach to obtain natural products. In this work, enantiomerically pure (*R*)-(+)-δ-decalactone was obtained by reduction of the C=C double bond of natural massoia lactone in a continuous-flow reactor. Of 13 different ene-reductases isolated, purified and tested, OYE3 was found to be the most efficient biocatalyst. The selected biocatalyst, either in the form of purified enzyme, cell lysate, whole cells or immobilized cells, was tested in the batch system as well as in the packed-bed flow bioreactor. The biotransformation performed in batch mode, using Ca^2+^-alginate immobilized cells of *Escherichia coli* BL21(DE3)/pET30a-OYE3, furnished the desired product with complete conversion in 30 min. The process was intensified using a continuous-flow reactor-membrane filtration system (flow 0.1 mL/min, substrate concentration 10 mM, pH 7, 24 °C) with cell lysate as biocatalyst combined with a cofactor regeneration system, which allowed obtaining > 99% bioconversion of massoia lactone.

## Introduction

Increasing attention is being paid to the origin of food additives, and those with natural origin are preferred by customers. Extraction and isolation of natural food additives, especially flavors, is sometimes expensive. It is due to low concentration of the desired compounds contained in raw materials^1^. An alternative route for flavor synthesis is based on application of enzymes or whole cells, which permits transformation with high chemo-, regio-, and stereoselectivity. Compounds obtained by biotransformation of natural starting materials are regarded as natural, according to United States and European Union regulations; therefore, interest in the biotechnological production of natural flavor compounds has recently increased^2^.

One group of additives with well-characterized flavor properties are compounds that contain ester bonds, including lactones, which are characterized by an intense, specific aroma, and which are widely used in the food, cosmetic, and pharmaceutical industries. Their fragrance depends on the size of the ring, the type of substituents, the presence of unsaturated bonds, and the configuration of the stereogenic centers^3,4^. δ-Decalactone, with its creamy, coconut-like, sweet, milky, fruit flavor, is of great interest to the industry. It is used to augment and enhance the aroma and taste of food preparations, chewing gums, beverages, toothpastes, colognes, perfumes and detergents^5,6^. Many flavors and fragrances show optical activity. It is well-known that the odor quality of optically active substances is much superior than their racemic compounds. (*S*)-(–)-δ-Decalactone odor description is peach, fatty, and buttery, whereas (*R*)-(+)-δ-decalactone exhibits sweet, fruity and milky aroma properties and is used as additive in the production of cheese and butter^5^.

One of the precursors of (*R*)-(+)-δ-decalactone is (*R*)-(–)-massoia lactone, which is commonly obtained by extraction from *Cryptocaria massoia* tree bark with high enantiomeric purity (ee > 99%) or prepared by synthesis^7^. The reduction of the endocyclic C=C double bond conjugated with the electron-withdrawing ester group can be catalysed by flavin-dependent ene-reductases which belong to the Old Yellow Enzyme (OYE) family (EC 1.6.99.1). These enzymes are already well known as catalysts allowing to obtain chiral building blocks for organic chemistry applications^8–10^. The production of δ-decalactone from massoia lactone by utilizing the reducing power of yeast (*Saccharomyces cerevisiae*) and bacteria of the species *Pseudomonas*, *Proteus* and *Bacillus* have been reported in patents (EP0822259A1, US5763233) and in the literature^11,12^. However, this approach is limited by the difficulty of using high substrate concentrations and the necessity of long time of biotransformation. Recently, ene-reductases have become available in sufficient quantities through the use of molecular biology techniques. This has prompted the investigation of reduction capability of isolated enzymes, such as OYE1 from *Saccharomyces pastorianus* and OYE2 and OYE3 obtained from *S. cerevisiae*^13^. Worth to mention, there are no reports describing the application of isolated ene-reductases to reduce massoia lactone to δ-decalactone so far.

The key concepts of modern industrial biotechnology are green chemistry and process intensification. In this view, biocatalysis and continuous processing are among the most promising green research areas for sustainable manufacturing of food ingredients, pharmaceutical intermediates and fine chemicals. These two approaches can be combined by immobilization of the biocatalysts and their use in continuous-flow packed bed bioreactors.

Immobilization of catalyst can improve its stability, minimize product inhibition, and allow for continuous processing by enabling re-use. Moreover, immobilization facilitates the downstream procedures of separation and purification of the products^14^. Additionally, flow processing conducted in packed bed bioreactors has the potential to accelerate biotransformation due to enhanced mass transfer, making large-scale production more economically feasible with a considerable decrease in reaction time. It has the advantage of avoiding loss of enzyme and additional separation steps, preventing deactivation of the enzyme caused by mechanical damage compared with stirring tank reactor and space–time yield improvement. In literature there are the examples of applying continuous‐flow mode of packed‐bed enzyme reactors for the kinetic resolution of biologically active compounds^15–20^. One of the most common techniques used for catalysts immobilization is alginate gel entrapment, which is an effective and simple strategy due to its large capacity for immobilized biomass.

The main goal of study was to develop biocatalytic methods aimed at the preparation of industrially important aroma compound (*R*)-(+)-δ-decalactone from natural massoia lactone. In this work, selected ene-reductase (OYE3) in the form of purified enzyme, whole cells and immobilized cells expressing this enzyme were tested for massoia lactone reduction. Biocatalysis was performed in the batch system; however, to intensify this process the packed-bed flow bioreactor was applied. Continuous flow-based reduction with immobilized cells and lysate was performed under a segmented liquid–gas flow system.

## Results and discussion

### Ene-reductase screening

**Figure 1:**
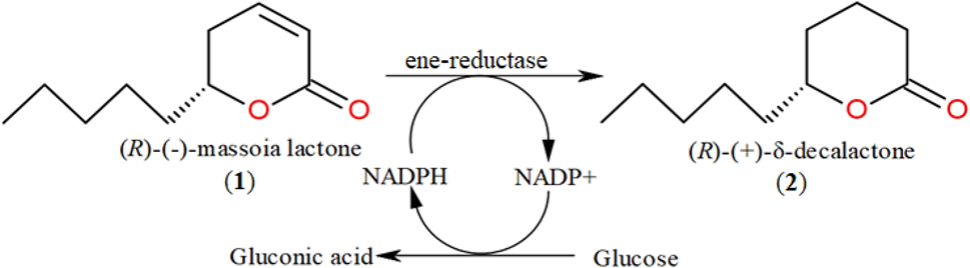
OYE-mediated reduction of massoia lactone (1) to (*R*)-(+)-δ-decalactone (2).

The first stage of the study was the selection of enzymes capable of reducing C=C double bond in (*R*)-(–)-massoia lactone (**1**) (Fig. 1), obtained by extraction from the *Cryptocaria massoia* tree bark. The regeneration of the NADPH cofactor was performed using a glucose dehydrogenase from *Bacillus megaterium*, with glucose as a co-substrate^21^.

**Figure 2:**
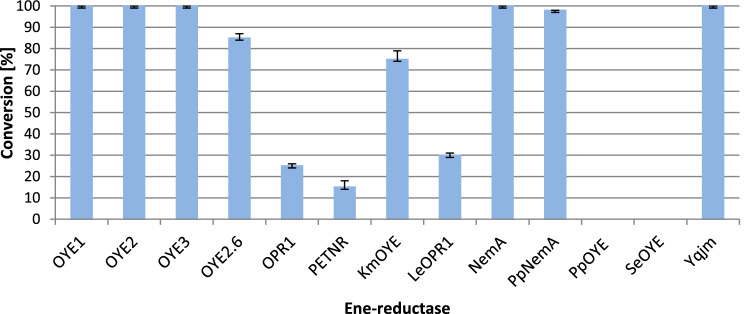
Results of massoia lactone (**1**) conversion by selected ene-reductases after 72 h (reaction conditions: 10 µL of 500 mM massoia lactone, 10 µL of 10 mM NADP^+^ solution, 20 µL of 1 M glucose solution, and 10 µL of GDH (5 mg/mL), ene-reductase: 100 µL (OYE1—50 µL, OYE2, OYE2.6, OYE3 and NemA—40 µL), 50 mM phosphate buffer pH 7–1 mL).

Selected enzymes of microbial and plant origin were obtained using the *Escherichia coli* expression system. Each of the 13 selected ene-reductases was isolated, purified, and tested for biotransformation of (*R*)-(–)-massoia lactone (**1**) to (*R*)-(+)-δ-decalactone (**2**). The degree of conversion was checked after 72 h (Fig. 2).

**Figure 3:**
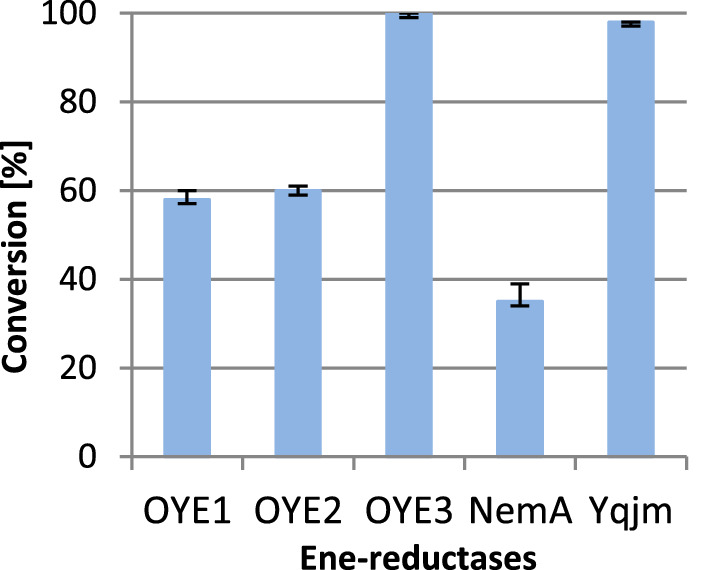
Results of massoia lactone (**1**) conversion by selected ene-reductases after 24 h (reaction conditions: 10 µL of 500 mM massoia lactone, 10 µL of 10 mM NADP^+^ solution, 20 µL of 1 M glucose solution, and 10 µL of GDH (5 mg/mL), ene-reductases: OYE1—5 µL, OYE2, OYE3 and NemA—4 µL, YqjM—10 µL, 50 mM phosphate buffer pH 7–1 mL).

Of the 13 ene-reductases tested, five of them catalyzed the total reduction of lactone **1** to the desired product **2**. In a further screening stage, it was decided to study the degree of substrate **1** conversion using tenfold less of selected enzymes (OYE1, OYE2, OYE3, NemA and YqjM) in the reaction mixture, and the biotransformation products were extracted after 24 h (Fig. 3).

The highest conversion (> 99%) of (*R*)-(*–*)-massoia lactone (**1**) was achieved when OYE3 was used as a biocatalyst, negligible lower conversion (98%) was observed with YqjM enzyme. Considering the obtained results, for further study of massoia lactone reduction OYE3 was chosen as a biocatalyst.

The substrate spectrum and stereoselectivity of yeast origin OYEs, among them OYE3, have been extensively studied in the last decade^10,17,21,22^. The advantages of using OYE-catalyzed hydrogenation for the synthesis of Active Pharmaceutical Ingredients (APIs) and fragrances have been demonstrated in several studies^23,24^. The OYE3 was obtained and purified for the first time by Niino et al. from yeast *S. cerevisiae* using *E. coli* expression system. The studies performed by his research group indicated that OYE3 (44,920 Da) has 73% similarity of DNA sequence and 89% similarity of amino acid sequence to OYE2. Despite the similarities, both enzymes exhibit different activities—OYE3 showed higher reducing activity^25^. Tasnádi et al. investigated various ene-reductases (among them OYE1–3, YqjM and OPR1), which have been studied for the bioreduction of cinnamic-ester derivatives. They established that OYE3 has ability to accept larger substrates due to the presence of serine (S297) in the binding pocket, whereas OYE1 and OYE2 have smaller binding pocket caused by the presence of phenylalanine (F296)^26^.

The use of isolated enzyme for the biotransformation of massoia lactone has significant advantages, because it avoids undesirable side reactions and ensures high yields. However, it requires many, sometimes expensive unit operations, such as subjecting cells to a disintegration process, and then purifying the desired enzymes by using chromatographic techniques or molecular filtration.

### Reduction of (*R*)-(–)-massoia lactone (**1**) in batch system with resting cells

The use of resting cells is an attractive solution, because this method requires the least number of unit operations. Additionally, there is no necessity to add to the reaction environment a cofactor and its regeneration system components, which are essential for an enzyme activity.

To investigate whether resting cells also catalyze the biotransformation of massoia lactone (**1**), reactions were carried out using different amount of *E. coli* BL21(DE3)/pET30a-OYE3 biomass (30 and 60 mg cells/mL reaction mixture) suspended in phosphate buffer (50 mM, pH 7.0) and different substrate **1** concentration (3 and 6 mM). Chromatographic analysis of the post-reaction mixture (after 24 h) showed the presence of only (+)-δ-decalactone ((+)-**2**) regardless of the applied biotransformation conditions. The structure of product was confirmed by ^1^H NMR and ^13^C NMR.

### Reduction of (*R*)-(–)-massoia lactone (**1**) in batch system with immobilized cells

In a further study, the strategy of whole-cell immobilization was employed to enhance the reusability of the biocatalytic system. Commonly applied methods include covalent linking to solid matrices, or entrapment and encapsulation in polymeric networks. Among these approaches, one of the most convenient methods for cells immobilization is encapsulation in alginate beads. Their mild gelation condition, high porosity and inert aqueous matrix help preserve the properties of the encapsulated biomass. However, dissolution of alginate beads was observed when the most convenient phosphate buffer (50 mM, pH 7.0) for OYE3 reduction was used as a reaction medium. In order to find the suitable conditions for immobilized *E. coli* BL21(DE3)/pET30a-OYE3 cells for the bioreduction of (*R*)-(–)-massoia lactone (**1**) to (*R*)-(+)-δ-decalactone (**2**), several approaches were applied. In the first one, biomass was suspended in either Tris–HCl (50 mM, pH 7.0) or sodium acetate buffer (20 mM, pH 6.0), immobilized using 4% solution of sodium alginate. The alginate gel beads (containing approximately 200 mg of cells) thus obtained were used as a biocatalyst in the wet and dried form.

The dissolution of alginate beads was not observed, when these buffers were used as a reaction environment. However, the results of biotransformation indicate the presence of desired product **2** merely when wet beads in Tris–HCl buffer were employed. The maximum conversion of (*R*)-(–)-massoia lactone (**1**) to **2** (85%) was achievedafter 96 h of reaction. Complete lack of the ene-reductase activity was observed when sodium acetate buffer was used.

Although dried alginate beads with entrapped microbial biomass and enzymes were successfully applied in other biotransformation processes^16,27^, in this work a complete loss of reduction activity of immobilized *E. coli* cells overexpressing the OYE3 enzyme was observed. Considering the obtained results, it was decided to continue experiments using immobilized *E. coli* cells in the form of wet beads in Tris–HCl buffer. To ensure better exchange of substrate **1** and product** 2** between the entrapped cells and the reaction environment, entrapment was performed using sodium alginate solution with twice lower concentration (2%). Additionally, to check the impact of glucose on the reduction of massoia lactone (**1**) to (+)-δ-decalactone (**2**), glucose (0.2% w/v) was added to the reaction mixture.

**Figure 4:**
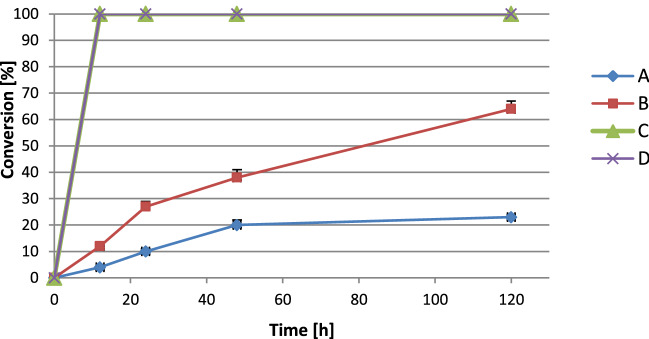
Time-course of the reduction of massoia lactone (**1**) with immobilized cells of *E. coli* BL21(DE3)/pET30a-OYE3 in batch mode. (**A**) 200 mg immobilized biomass without glucose, (**B**) 400 mg immobilized biomass without glucose, (**C**) 200 mg immobilized biomass with glucose, (**D**) 400 mg immobilized biomass with glucose.

The results presented in Fig. 4 (case **C** and **D**) show that the addition of glucose definitely improves massoia lactone (**1**) reduction process, probably due to the fact that it is an essential substrate for NADP^+^ regeneration (Fig. 1) or the addition of glucose probably led to a metabolic boost affecting positively the efficiency of cofactor regeneration. In these cases, the complete transformation of substrate **1** was determined in the first sample collected after 12 h of biotransformation. Additionally, the lower degree of entrapment accelerated the biotransformation process.

The immobilization of biocatalyst was also performed using agar–agar for entrapment. The analysis indicated that the complete conversion of (*R*)-(*–*)-massoia lactone (**1**) to δ-decalactone (**2**) was achieved after 12 h of reaction using agar–agar cubes with immobilized biomass of *E. coli* BL21(DE3)/pET30a-OYE3. The investigation on OYE3 immobilization was conducted by Tentori et al., where OYE3 was immobilized both by covalent binding on glyoxyl-agarose (GA), and by affinity-based adsorption on EziGTM particles^17^. The OYE3/GDH-EziG (co-immobilized OYE3 and GDH) exhibit lower bioreduction activity against α-methyl-*trans*-cinnamaldehyde than OYE3-GA, which was probably related to the loss of immobilized GDH activity during the biotransformation process.

Both the biocatalyst in the form of Ca^2+^-alginate beads and agar–agar cubes showed similar reducing activity against substrate (**1**). Considering the convenience of obtaining the biocatalyst in the form of Ca^2+^-alginate spheres, it was decided to continue the research by immobilizing *E. coli* BL21(DE3)/pET30a-OYE3 (co-expressing OYE3 and GDH) using this technique.

**Figure 5:**
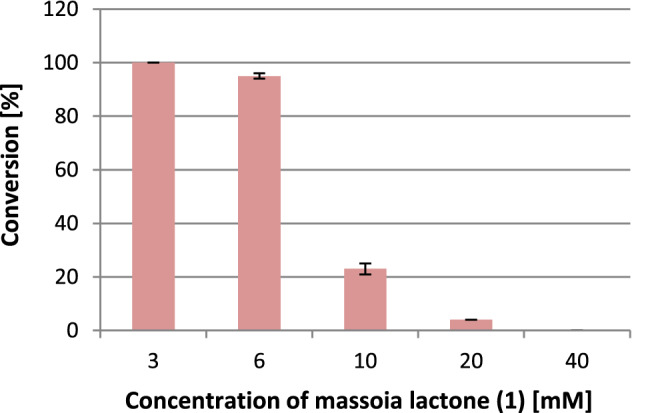
Conversion of massoia lactone (**1**) with immobilized cells of *E. coli* BL21(DE3)/pET30a-OYE3 in batch mode with different concentration of substrate **1** (4 h reaction time).

Next experiments were performed in batch conditions with different massoia lactone concentration. Beads with entrapped *E. coli* BL21(DE3)/pET30a-OYE3 cells containing approximately 200 mg of wet biomass were introduced into 20 mL glass vials and a solution consisting of a mixture of Tris–HCl buffer, glucose (0.2% and 0.5% w/v) and substrate **1** (3–40 mM) in a final volume of 5 mL was added. The Fig. 5 shows results of 4-h reaction where the highest conversion (> 99%) was achieved in the batch with the 3 mM concentration of substrate **1**), whereas the use of a 10 mM massoia lactone (**1**) solution reduced the conversion by 5 times.

### Flow biotransformation with immobilized cells

**Figure 6:**
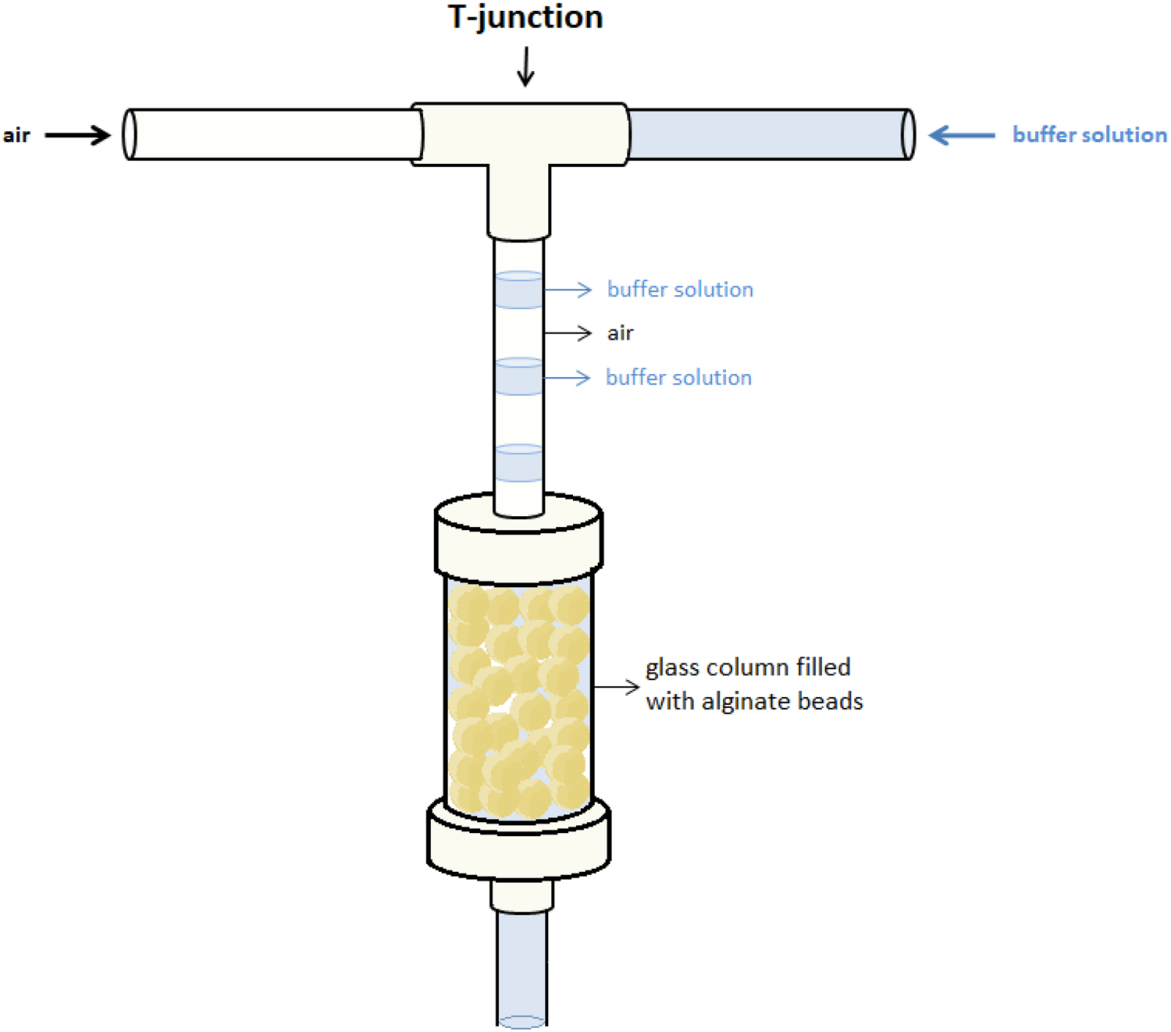
Scheme of packed-bed bioreactor with the segmented gas–liquid flow applied in experiment.

The reduction process of massoia lactone (**1**) was also tested under continuous-flow conditions, by the application of a packed-bed bioreactor. Alginate beads with immobilized *E. coli* BL21(DE3)/pET30a-OYE3 cells (3.5 g) were loaded onto a glass column (i.d. 15 mm, length 3 cm). A first set of experiments was carried out by simply feeding the packed bed bioreactor with massoia lactone (**1**) (3 mM) and glucose (0.5% w/v) solution in Tris–HCl buffer at different flow rates; however, no biotransformation was observed, regardless of the set flow. Therefore, it was decided to supply oxygen to the reaction environment by a segmented gas–liquid flow. Providing oxygen to the immobilized cells supported their vital functions, enabling the reduction of massoia lactone (**1**). The gas phase and the liquid phase were merged in a T-junction (air flow: 100 µL/min, liquid flow: 100 µL/min, residence time: 25 min), allowing to generate air–buffer solution segments in the flow stream entering the packed-bed reactor (Fig. 6). Flow biotransformation of substrate **1** was also conducted using cells entrapped in agar–agar as biocatalysts.

**Figure 7:**
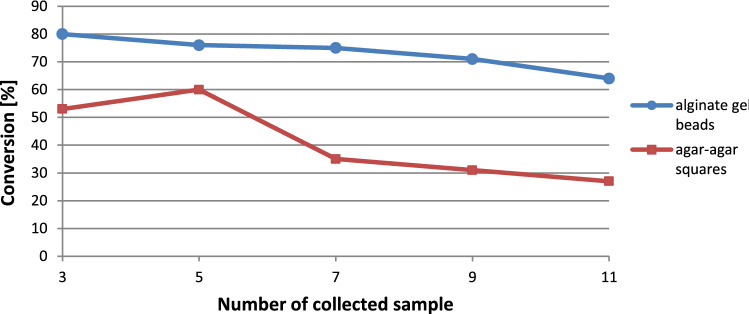
Conversion of massoia lactone (**1**) with immobilized cells of *E. coli* BL21(DE3)/pET30a-OYE3 in semi-continuous flow system with the use of segmented gas–liquid flow.

The application of flow system avoids problems related to substrate and product inhibition impact on immobilized biocatalyst; however, the complete conversion to desired product **2** was not achieved and the reduction activity exhibited by immobilized *E. coli* cells decreased during semi-continuous operation (Fig. 7). This could be due to partial damage of the immobilized system, the NADP^+^ cofactor that participates in the reduction system may be lost by cells damaged by shear stress occurring in flow reactors^16^.

### Flow biotransformation with cell lysate

**Figure 8:**
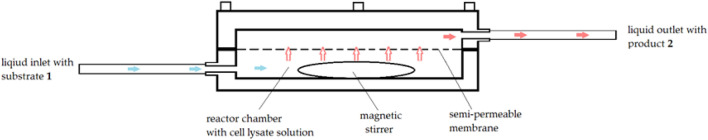
Scheme of reactor system with a semi-permeable membrane between cell lysate and massoia lactone (**1**) solutions.

Finally, it was decided to use the biocatalyst in the form of the *E. coli* BL21 (DE3) pET30a-OYE3 cell lysate obtained by sonication. The use of cell lysate allows the cell-reaction environment barrier to be bypassed, ensuring better access of the enzyme to the substrate. The reduction of massoia lactone (**1**) was conducted in flow reactor with a membrane system. The semi-cellulose membrane constituted a semi-permeable barrier, retaining the enzymes contained in the cell lysate and letting a biotransformation product **2** (δ-decalactone) passthrough the system. The membrane could retain both OYE3 and GDH, allowing the continuous use of the whole recycling system and saving NADP^+^ that flew through reactor with glucose and the substrate **1** solution (Fig. 8).

The solution of 10 mM massoia lactone (**1**) was pumped at a constant flow rate of 100 µL/min in thermostatic condition through a semi-cellulose membrane placed in the membrane reactor containing the solution of lysate and cofactor regeneration system. The residence time was 120 min. In the analysed samples the presence of (*R*)-(+)-δ-decalactone (**2**) was detected. The outlet conversion was constant (> 99%), no decrease in the reducing activity of the catalyst was observed until the end of the flow process (5 h). The space–time yield (STY) for continuous-flow reduction with lysate was calculated as below:

$${STY}_{flow}=\frac{{n}_{reagent}\cdot C}{{V}_{lysate}\cdot \tau }=\frac{0.3\cdot 99}{0.002\cdot 2}=7.425 \mathrm{M }{\mathrm{h}}^{-1} {\mathrm{L}}^{-1}$$

where n_reagent_ (mmol)—amount of reagent; *C* (%)—conversion of the reagent into the desired product determined by GC/MS analysis; V_lysate_ (L)—volume of lysate solution; *τ* (h)—residence time^28^

## Materials and methods

### General

Chemicals and solvents were purchased from Merck (Milano, Italy) and used without further purification. (*R*)-(–)-Massoia lactone (**1**) from the *Cryptocaria massoia* tree bark was provided by prof. Claudio Fuganti (Politecnico di Milano, Italy). Substrate was used for biotransformation as a 500 mM solution in DMSO. Ni Sepharose™ 6 Fast Flow gel was purchased from GE Healthcare BioSciences. GC–MS analyses were performed using a HP-5MS column (30 m × 0.25 mm × 0.25 µm, Agilent). The following temperature program was employed: 60 °C (1 min), 6 °C min^−1^/150 °C (1 min), 12 °C min^−1^/280 °C (5 min). Bradford assay was performed by using a Shimadzu UV-1601 spectrophotometer and bovine serum albumin (BSA) was used as reference standard protein^17^. All the enzymatic reactions were kept under stirring and controlled temperature by using a thermoshaker New Brunswick—Excella^®^ E24. Reaction extraction and phase separation were accomplished through a ZX3 Advanced Vortex Mixer from VELP Scientifica and a Microcentrifuge ScanSpeed Mini (LaboGene). The continuous flow reactions were performed using an E-Series Integrated Flow Chemistry system from Vapourtec (Alfatech S.P.A., Genoa, Italy) equipped with Omnifit glass columns (15 mm i.d. × 100 mm length). As membrane reactor, an ultrafiltration chamber was employed (63.5 mm i.d.). Ultrafiltration chamber was equipped with a magnetic stirrer reducing the available volume from 20 mL (empty volume) to 12 mL (effective volume). Membranes for ultrafiltration (regenerated cellulose membranes, i.d. 63.5 mm, cut-off 5 kDa) were purchased from Merck^29^. The ^1^H NMR and ^13^C NMR spectra were recorded with a Varian Mercury 300 (300 MHz) spectrometer.

### Enzyme production

GDH from *Bacillus megaterium* and tested ene-reductases and were prepared as His-tagged proteins (name, UniProtKB accession number): OYE1, Q02899; OYE2, Q03558; OYE3, P41816; PETNR, P71278, and GST-tagged proteins: OYE2.6, A3LT82; KmOYE, Q6I7B7; OPR1, Q8LAH7; LeOPR1, Q9XG54; NemA, P77258; PpNemA, Q88I29; SeOYE, Q31R14; PpOYE, Q88K07, non-tagged: YqjM, P54550. Ene-reductases were purified according to standard methods^22^.

### Screening of ene-reductases

In a 2 mL test tube, a solution of massoia lactone (**1**) (500 mM in DMSO) was added to 50 mM phosphate buffer pH 7, followed by 10 µL of NADP^+^ solution (10 mM), 20 µL of glucose solution (1 M), and 10 µL of GDH (5 mg/mL). The reaction was started by adding different volumes of selected ene-reductases, according to their different concentrations, in order to obtain a final concentrations of 100 µg/mL in the reaction (the final volume of reaction solution: 1 mL) and stirred in the thermoshaker at 28 °C for 72 h. The reaction was stopped by adding to reaction mixture 400 µL of ethyl acetate and vortexing by 30 s. The organic phase was separated by centrifugation (2 min, 13,500 rpm), dried over Na_2_SO_4_ for 5 min, and analyzed by GC–MS (see “General” for analytical conditions). The conversion was evaluated from the ratio of the peak areas of product and substrate. All the biotransformation experiments were carried out in triplicate. Statistical analysis indicated that variations between obtained data were non-significant.

### Biomass preparation

*Escherichia coli* BL21(DE3)/pET30a-OYE3 (stored in − 80 °C) was inoculated on Petri plate containing solid LB medium (yeast extract 5 g/L, tripton 10 g/L, NaCl 10 g/L agar 15 g/L) with 100 mg/mL kanamycin (kan_100_) and grown overnight at 37 °C. Obtained culture was used to inoculate 100 mL of liquid LB medium (kan_100_) in 350 mL Erlenmayer flask (37 °C, 19 h, 200 rpm). 30 mL of obtained culture was used to inoculate 750 mL LB medium (kan_100_) in 4 L Erlenmayer flask and incubated at 37 °C, 200 rpm. In order to induce of OYE3 enzyme overexpression, 150 µL of 0.5 M IPTG was added to Erlenmayer flask, when OD_600_ of culture was in the range of 0.45–0.5. Incubation was continued for the next 4 h at 30 °C (220 rpm). The weight of wet cells harvested by centrifugation (5000 rpm, 30 min, 4 °C) ranged from 6.5 to 7.5 g.

### Cell immobilization

Obtained biomass of *E. coli* BL21(DE3)/pET30a-OYE3 (see “Biomass preparation”) was washed with NaCl solution (0.9% (w/v)), divided in two parts, and then suspended into 50 mL of Tris–HCl buffer (50 mM, pH 7.0) and 50 ml of sodium acetate buffer (20 mM, pH 6.0). Alginate gel beads were formed by dripping the mixture of obtained cell suspensions and 50 mL of 4% (w/v) sodium alginate into 500 mL of 0.2 M CaCl_2_ solution kept under gentle magnetic stirring with subsequent solidification for 20 min. The formed beads were then rinsed with deionized water, weighted, and preserved at 4 °C until use. Half of obtained beads were dried at 25 °C for 16 h. Alginate beads showed spherical structures with diameters of 4.0 ± 0.3 mm, whereas dried beads showed sizes ranging from 1.6 to 2.2 mm.

The entrapment of cells within agar–agar carrier was initiated by mixing cells suspension with agar–agar solution in a ratio of 1:3. First, 2.0% of agar–agar solution was prepared in 0.9% (w/v) NaCl solution and dissolved by continuous shaking at 100 °C. The solution was cooled down between 30 and 40 °C. Then, the obtained *E. coli* biomass (see “Biomass preparation”) was suspended in 5 mL of 0.9% (w/v) NaCl and mixed thoroughly in the 15 mL agar–agar solution and poured immediately on Petri plate (Ø 6 cm). The solidified gel of agar–agar with entrapped cells was cut into equal sizes of 4.0 × 4.0 mm and washed with deionized water. Prepared squares were weighted and stored at 4 °C.

### General procedure for batch (*R*)-(–)-massoia lactone (**1**) reduction with resting cells

1 mL suspension of *E. coli* BL21(DE3)/pET30a-OYE3 containing 0.3 and 0.6 mg of cells/mL of phosphate buffer (50 mM, pH 7.0) with 0.2% (w/v) of glucose was prepared in the 1.5 mL of Eppendorf tubes. Then, substrate **1** was added to obtain 3 mM and 6 mM solutions and reactions were maintained at 30 °C with continuous shaking (150 rpm) for 24 h. In order to assess progress of massoia lactone (**1**) reduction, samples (0.3 mL) were collected and extracted by vortexing (13,500 rpm, 30 s) using 0.3 mL of ethyl acetate. Finally, the organic phase was dehydrated by anhydrous Na_2_SO_4_ and transferred to a vial, then analysed on a GC–MS instrument equipped with an autosampler (see “General”).

### General procedure for batch (*R*)-(–)-massoia lactone (**1**) reduction with immobilized cells

A 3 mM solution of massoia lactone (**1**) with 0.2% (w/v) glucose was prepared in 5 mL of Tris–HCl buffer (50 mM, pH 7.0). The reaction solution was pre-incubated in 20 mL vials at 30 °C for 5 min. Then, carrier with immobilized biomass (3.5 ± 0.05 g) was added and reaction was maintained at 30 °C with continuous shaking (150 rpm). In order to assess progress of massoia lactone (**1**) reduction in time, samples (0.3 mL) were collected and extracted by vortexing (13,500 rpm, 30 s) using 0.3 mL of ethyl acetate. Finally, the organic phase was dehydrated by anhydrous Na_2_SO_4_ and transferred to a vial then analysed on a GC–MS instrument equipped with an autosampler.

### General procedure for flow (*R*)-(–)-massoia lactone (**1**) reduction with immobilized cells

The flow-reactor system (Vapourtec E-Series pump) consisted of a 1.5 cm internal diameter plastic glass column packed with alginate gel beads (3.5 ± 0.05 g) of 4 ± 0.2 mm in diameter (length of reactor filling—3 cm). Then, a buffer solution of massoia lactone (**1**) (3 mM in Tris–HCl 50 mM, pH 7.0) was flowed through the column at a constant flow rate of 100 µL/min in thermostatic condition of 30 °C with a residence time of about 25 min. Air was delivered at a flow rate of 100 µL/min by joining the airflow at the T-junction, before entering the column. Samples containing final reaction mixture (~ 1.5 mL each) were collected in 2 mL Eppendorf tubes. Subsequently, 0.5 mL of sample was taken and extracted with 0.3 mL of ethyl acetate by vortexing (13,500 rpm, 30 s). The organic phase was dehydrated by anhydrous Na_2_SO_4_, transferred to a vial and analysed on a GC–MS instrument.

### Cell lysate preparation

*Escherichia coli* BL21(DE3)/pET30a-OYE3 cells (see “Biomass preparation”) were recovered by centrifugation and washed three times with sterile distilled water to remove possible organic contamination. Then, 1 g of the pellet was suspended in 5 mL of phosphate buffer (50 mM, pH 7.0) and sonicated for 7 consecutive times for 20 s at 1 min intervals with gentle mixing after every 2 cycles. The cell debris was removed by centrifugation (10,000 rpm, 30 min, 4 °C) and the supernatant was stored at − 80 °C.

### General procedure for flow massoia lactone (1) reduction with cell lysate

The reactor system consisted of a Vapourtec E-Series pump with a membrane reactor. The solution of 10 mM massoia lactone (**1**) with 600 µL of glucose (1 M) and 300 µL of NADP^+^ (10 mM) in phosphate buffer (50 mM, pH 7.0) for a final volume of 30 mL was pumped through a stirred membrane reactor (12 mL reaction volume, i.d. 63.5 mm, 5 kDa membrane cut-off) containing 150 µL of GDH (5 mg/mL) and 2 mL *E. coli* BL21(DE3)/pET30a-OYE3 cell lysate at a constant flow rate of 100 µL/min (120 min residence time) in thermostatic condition of 24 °C for 5 h. Samples containing final reaction mixture (~ 1.5 mL each) were collected in 2 mL Eppendorf tubes. Subsequently, 0.5 mL of sample was taken and extracted with 0.3 mL of ethyl acetate by vortexing (13,500 rpm, 30 s). The organic phase was dehydrated by anhydrous Na_2_SO_4_, transferred to a vial and analysed on a GC–MS instrument.

## Conclusion

The studies involved the production and selection of various forms of OYE3 which was selected during the screening process among 13 isolated, purified and tested ene-reductases applied in the bioconversion of massoia lactone (**1**) to δ-decalactone (**2**). This biocatalyst was used in the form of a purified enzyme, resting cells, entrapped in Ca^2+^-alginate and agar–agar, and the form of a cell lysate. To compare and improve the efficiency of the process, bioreduction was carried out in both batch and flow conditions. Biocatalyst in the form of alginate beads provided desired product **2** in 30 min in the batch condition; however, during the biotransformation with the immobilized cells in the continuous-flow system, a decrease in conversion with the duration of the process was observed. The problem was solved by applying a system comprising of a continuous-flow membrane reactor containing cell lysate, through which the solution with substrate **1** and a gaseous stream of pressurized air flowed. This solution proved to be highly applicable to the bioreduction of massoia lactone with > 99% conversion rate. In the longer term, activities are planned to increase the efficiency of the process for obtaining δ-decalactone (**2**) from massoia lactone (**1**) using a selected biocatalyst.
